# Hormone induced differential transcriptome analysis of Sertoli cells during postnatal maturation of rat testes

**DOI:** 10.1371/journal.pone.0191201

**Published:** 2018-01-17

**Authors:** Mukesh Gautam, Indrashis Bhattacharya, Umesh Rai, Subeer S. Majumdar

**Affiliations:** 1 Department of Zoology, University of Delhi, Delhi, India; 2 Cellular Endocrinology Laboratory, National Institute of Immunology, New Delhi, India; 3 National Institute of Animal Biotechnology, Hyderabad, India; University of Hyderabad, INDIA

## Abstract

Sertoli cells (Sc) are unique somatic cells of testis that are the target of both FSH and testosterone (T) and regulate spermatogenesis. Although Sc of neonatal rat testes are exposed to high levels of FSH and T, robust differentiation of spermatogonial cells becomes conspicuous only after 11-days of postnatal age. We have demonstrated earlier that a developmental switch in terms of hormonal responsiveness occurs in rat Sc at around 12 days of postnatal age during the rapid transition of spermatogonia A to B. Therefore, such “functional maturation” of Sc, during pubertal development becomes prerequisite for the onset of spermatogenesis. However, a conspicuous difference in robust hormone (both T and FSH) induced gene expression during the different phases of Sc maturation restricts our understanding about molecular events necessary for the spermatogenic onset and maintenance. Here, using microarray technology, we for the first time have compared the differential transcriptional profile of Sc isolated and cultured from immature (5 days old), maturing (12 days old) and mature (60 days old) rat testes. Our data revealed that immature Sc express genes involved in cellular growth, metabolism, chemokines, cell division, MAPK and Wnt pathways, while mature Sc are more specialized expressing genes involved in glucose metabolism, phagocytosis, insulin signaling and cytoskeleton structuring. Taken together, this differential transcriptome data provide an important resource to reveal the molecular network of Sc maturation which is necessary to govern male germ cell differentiation, hence, will improve our current understanding of the etiology of some forms of idiopathic male infertility.

## Introduction

Spermatogenesis is a complex process where every step of male Germ cell (Gc) development are essentially supported by somatic Sertoli cells (Sc) [1]. In response to various hormonal and biochemical stimulation Sc produce important factors that regulate Gc division and differentiation [1,2]. The functions of Sc are largely governed by the synergistic effect of Follicle Stimulating Hormone (FSH) and testosterone (T) as Sc bear receptors for both the hormones [3]. Although Sc of infant primates and rodents are exposed to sufficient levels of FSH and T, robust onset of spermatogenesis is not seen during infancy but is discernible only during the onset of puberty [4,5]. We have demonstrated earlier that a developmental switch, in terms of hormonal responsiveness, of Sc occurs in rats at around 12 days of postnatal age coinciding with the rapid transition of spermatogonia A to B [4]. During pubertal development, Sc become mature further with the changing need of differentiating Gc to support spermatogenesis. Therefore, such a pubertal maturation of testicular Sc is considered to be a prerequisite of male fertility [6].

In rodents, upto 3–5 days after birth, Sc keep proliferating and attract the gonocytes towards the basement membrane for establishing the spermatogonial stem cell (SSC) niche [7]. Neonatal Sc continue to proliferate and thereby directly regulating the numbers of SSC niches in the developing testes [8]. During second week of postnatal life, as the Gc enter into meiosis, Sc stop proliferating and become enlarged to hold the growing number of Gc within their cytoplasmic extensions. The Sc-Sc tight junctions are formed to establish the blood-testis-barrier (BTB) at the age of 15–16 days after birth [9]. Sc prominently expresses tight junction and adherent junction proteins to hold various stages from spermatocyte to elongated spermatids. Besides nurturing, mature Sc also maintain a finite number of Gc by regulating apoptosis, scavenging dead cells, and changing adherent junctions to release mature sperm into lumen. By the age of 55–60 days, fully mature sperm are seen in epididymis of rat suggesting that by this time, adult Sc are fully differentiated to extend support to all stages of Gc for maintaining spermatogenesis [10].

To understand the regulatory mechanism of spermatogenesis, it is imperative to explore the changing landscape of gene expression during various phases of Sc maturation. Microarray is a powerful technique to study differentially expressed genes in large numbers [11]. Differential gene expression during spermatogenesis has been studied by various researchers using microarray technology studied by us [12,13] and others [14–16]. These gene expression profiling experiments reveal candidate genes for the regulation of spermatogenesis and fertility as well as targets for innovative contraceptives that act on gene products absent in other somatic tissues. Here, using microarray technology, we have compared the hormone stimulated (FSH and T in combination) differential transcriptome profile of Sc purified and cultured from immature (5 days old), maturing (12 days old) and mature (60 days old) rat testes. Our results suggest that transcriptome of Sc during different maturation stages differ significantly and this information can help advance our understanding of regulatory mechanisms of spermatogenesis.

## Material and methods

### Animals

Wistar outbred rats (*Rattus norvegicus*) were procured from colony maintained by Small Animal Facility, National Institute of Immunology, New Delhi, India. Animals were maintained in a standard day night cycle with stable temperature and humidity and provided food and water *ad libitum*. All the animal experimentations were approved by Institutional Animal Ethics Committee (IAEC) and performed following standard guidelines of ‘‘Committee for the Purpose of Control and Supervision of Experiment on Animals (CPCSEA),” Government of India.

### Isolation and culture of Sertoli cells from 5 days and 12 days old rat testes

Sc cultures were prepared from 5 days and 12 days old rat testes as described earlier [4]. Briefly, testes were decapsulated and chopped finely before sequential digestion with collagenase and pancreatin enzyme at 34°C. Clusters of Sc were separated from mixture of cell suspension by differential centrifugation. Cultures were maintained in DMEM-F12 HAM media containing 1% FBS for first 24 hrs before replacing with growth factor media (GF media) containing 5 μg/ml sodium selenite, 10 μg/ml insulin, 5μg/ml transferrin, and 2.5 ng/ml epidermal growth factor. Sc cultures were maintained in defined GF media for 4 days. On day 3, cells were treated with 20 mMTris·HCl (pH 7.4) for 3–5 min to get rid of contaminating Gc [17]. *In vitro* hormone treatment was performed on day 4.

### Isolation and culture of Sertoli cells from 60 days old rat

Sc from mature rat testes (60 days old) were isolated and cultured as described earlier [18]. Briefly, testes were decapuslated and seminiferous tubules were chopped, washed and repeatedly digested with 1mg/ml collagenase until most of Sc clusters were released. Sc cells were cultured and maintained for 4 days before initiation of experiments as described previously [4]. On day 3, cells were treated with 20 mMTris·HCl (pH 7.4) for 5 min to get rid of contaminating Gc [17].

### Purity of Sc culture

On day 4, Gc contamination were found to be less than 5% in Sc cultures of all age groups. Purity of Sc in culture were identified by vimentin staining (Abcam, USA, Ab8978) whereas Peritubular Cell (PTc) or Leydig Cell (Lc) contaminations were determined by the alkaline phosphatase or the 3β-HSD activity respectively, as described by us before [4,18].

### *In vitro* hormone treatment

On day 4 of culture, cells of all age groups were incubated with GF media containing hormones (50ng/ml o-FSH and 10^−7^M T in combination = FT media) in pulsatile manner (30min of FT pulse/3hr)The doses of both o-FSH and T were previously found bioactive in cultured Sc obtained from all three age groups studied [4,18]. Sc were exposed for ½ hour (hr) to FT media and then the FT media was removed and replenished with fresh GF media for 2^½^ hr. This was repeated upto 11 hr. Please note that since the treatment was terminated at 11^th^hr i.e. 1^1/2^ after receiving the 4^th^ pulse of FT media, Sc were in GF media at the time of termination. This experiment was repeated at least three times in each age group of Sc cultures prepared on different calendar dates. At the end of the experiments, cells were dislodged, washed and then suspended in RNA-later and stored at -80°C until RNA extraction.

### RNA extraction, labeling and microarray hybridization

Total RNA was isolated from Sc using Qiagen (Valencia, CA) RNAEasy Mini kit according to the instructions of the manufacturer. Purity of Total RNA was assessed by the NanoDrop^®^ ND-1000 UV-Vis Spectrophotometer (Nanodrop technologies, Rockland, USA). Total RNA with OD260/OD280>1.8 and OD260/OD270 ≥ 1.3 was used for microarray experiments. RNA integrity was assessed using RNA 6000 Nano Lab Chip on the 2100 Bioanalyzer (Agilent, Palo Alto, CA). For the assessment of total RNA quality, the Agilent 2100 Expert Software used to determine RNA Integrity Number (RIN) which provided a quantitative value for RNA integrity. RNA having RIN ≥9.0 out of maximum scoring of 10 was used for microarray hybridization. The RNA which passed quality control parameters was labeled with Cy3 dye and hybridized on rat whole genome 4X44K gene chip (Agilent Technology Inc.) as described by us earlier [19].

### Microarray scanning, feature extraction and array analysis

Hybridized arrays were scanned at 5μm resolution on an Agilent DNA Microarray Scanner, Model G2565BA. Data extraction from images was done using Feature Extraction software of Agilent. Hybridization signals were quantified using GeneSpringGx v 11.0.1 software from Agilent Technologies. Pearson’s correlation coefficients were computed to assess the reliability of data obtained from RNA preparation for 3 samples each from 5 day, 12 day and 60 day Sc. The data retrieved from separate membranes with the same RNA samples yielded QC statistics highly concordant with that of the manufacturer, and it revealed more than 95% confidence level. In order to identify biological variation, integrated signal analysis for a given membrane was performed and signal spots that were low after averaging, as compared to average background plus 2SD values, were removed. Within each hybridization panel, the 50th percentile of all measurements was used as a positive control for normalization for each gene. Data normalization, averaging, calculation of relative abundance of transcripts, ratio analysis, and fold changes were performed on log transformed data using GeneSpringGx v.11.0.1 (Agilent Technologies, Santa Clara, CA, USA). Per-membrane and per-gene normalization were conducted using GeneSpringGx v 11.0.1 normalization algorithms. Principal Component Analysis (PCA) was performed for all annotated 5 day, 12 day and 60 day Sc samples for all expressed genes to assess the similarity in gene expression patterns on the basis of underlying variability and cluster structures using algorithm in Gene- Spring11.0.1 [20].

K-means cluster analysis and unsupervised hierarchic clustering analysis (HCA) was performed using GeneSpringGx v.11.0.1 software. In K-means cluster analysis, gene expression levels were randomly assigned into distinct clusters and the average expression vector was computed for each cluster. For every gene, the algorithm then computed the distance to all expression vectors, and moved the gene to the cluster whose expression vector was closest to it. The entire process was repeated iteratively until no gene products could be reassigned to a different cluster. To further evaluate the patterns in gene expression profiles, unsupervised hierarchic clustering (HCA) of data in pairwise comparisons among samples using a standardized Pearson’s uncentered correlation vector with average linkage for distance measures, and their visualization in the form of a heat map and dendrogram were performed.

Normalized data were used in pairwise overall comparisons between 5 day, 12 day and 60 day samples. The resulting gene lists from each pairwise comparison only included the genes that showed a fold change of 2.0 or higher and a p< 0.05 by using a parametric Welch t test with Benjamini-Hochberg multiple testing corrections for false discovery rate (FDR). All statistical analyses were performed using GeneSpring v.11.0.1 software. Identification of differentially enriched gene ontology (GO) terms for genes in 4 clusters in K-means analysis, as well as, those identified in differential expression analysis was carried out using a gene ontology (GO) tree machine and GeneSpring v.11.0.1. Further, functional analysis of differentially expressed genes was performed using GeneGo Metacore software (Thermo scientific, St. Joseph, MI, USA), DAVID online data analysis tool and the Kyoto Encyclopedia of Genes and Genomes (KEGG) platform (http://www.genome.jp/kegg/) and STRING database for pathways analysis to link genomic information with higher order functional information. For preparation of heat maps of some important differentially expressed genes, a web based application matrix2png was used [21]. For making interacting network of genes, STRING database tool was used [22].

### Quantitative RT-PCR

Quantitative RT-PCR (qRT-PCR) amplifications were performed in additional sets of Sc cultures obtained from all 3 age groups with uniform hormonal treatments used in microarray to validate expression level of various genes as per described by us earlier [4,19,23] Dissociation curve analysis was performed immediately after amplification to ensure that there was only one (gene specific) amplification peak. For each sample, the calculated quantity of each gene was then normalized with relation to the quantity found for Ppia (Cyclophilin A). The relative quantities of mRNA for target genes were determined by 2^-ΔΔCt^ method. The means (±SEMs) of 3 individual experiments were determined for each treatment group for the target gene. The list of primers used for Real Time PCR is given in Table 1.

**Table 1:** Primers used for validation of microarray genes.

Gene Name	Accession Number	Primer Sequence		length	Tm °C	Product Size (bp)
AaSS	NM_013157.3	F	ATACAATGAAGAGCTGGTGAG	21	58.5	149
		R	GCCTCTTTGTCACGGTCTA	19	59.3	
ABP	NM_053706.1	F	AGGGTTTGCTGATTTTGGTG	20	60	129
		R	GACGGACCCTGAGACACATT	20	60	
Ccl5	NM_031116.3	F	GCTTTCCTGTCATTGCTTGC	20	64.9	136
		R	AGGCCATAGGAGAGGACACA	20	63.2	
Cldn 11	NM_053457.2	F	ACGGTTGCGTATGCTTTGA	19	60	131
		R	ACACCCATGAAGCCAAATT	19	60	
GJA1	NM_012567.2	F	GTCTACCCCTCTGGGTGTGA	20	60	180
		R	AGGACCAGTCGAGGATGATG	20	60	
Dmrt1	NM_015826.5	F	GGTCAGAGCATGTCCCAGAT	20	60	182
		R	GGTTCAGAGGACGCAGACTC	20	60	
GDNF	NM_019139.1	F	GGCCGACAATGTACGAC	17	60	172
		R	CCACACCGTTTAGCGGAA	18	60	
Ocln	NM_031329.2	F	CCCAGGTGGCAGGTAGATTA	20	60	193
		R	GCACCACGTTGGAAAAGAAT	20	58	
Ppia	NM_008907.1		ATGGTCAACCCCACCGTGT	19	60	101
			TCTGCTGTCTTTGGAACTTTGTCT	24	60	
PWWP1	NM_133549.2	F	GGCTCCCAAGTCATAAGATC	20	60.2	126
		R	TCAAAGCAGCAGCAGAAGTC	20	62.7	
ROBO1	NM_022188.1	F	GAGTATGCGGGCCTGAAG	18	62.3	147
		R	GGGTCTGGCTTTCTGGATTA	20	63.5	
SCF	NM_021843.4		GTGGATGACCTCGTGGCATGTA	20	60	155
			TCAGATGCCACCATGAAGTCC	21	60	
Spz1	NM_001024297.1	F	CGGAAGCAGAAAAGATGGAC	20	63.9	125
		R	GCGGTTATTTCGAGCCTTCT	20	65.9	
Testin	NM_173132.1	F	GGGTCATTGTGCCTCTAGTTG	21	63.8	117
		R	CCCATGCAGTCCAGTAGGTT	20	63.7	
Tf	NM_001013110.1	F	TCTGTTTGTTCCGGTCTTCC	20	60	203
		R	GCACCCACCTCTTGGATTT	19	60	
Fat3	NM_138544.1	F	GCCCAACTATGAGAGCCAAG	20	64.0	150
		R	GGGGGTAGCTGATCCTGACT	20	64.5	
Msln	NM_031658.1	F	GGAGGCTTGTGTCGATGGTA	20	64.0	137
		R	TCAGGGACTCGGGATAGCC	19	66.0	
Unc5c	NM_199407.1	F	TTAGCCAAGTTGCAGGGAAT	20	64.4	146
		R	CAAGGAGGAAGATGACTGGTT	21	62.4	
Wisp1	NM_031716.1	F	AAAGTCGCCTCTGCAACCT	19	63.7	120
		R	AGCCTGCGAGAGTGAAGTT	19	60.9	

### Statistical analysis

The differential expression of genes was determined by pairwise comparison of the genes that showed a fold change of 2.0 or higher and a p< 0.05 by using a parametric Welch t test with Benjamini-Hochberg multiple testing corrections for false discovery rate (FDR). The statistical analyses to generate gene expression list were performed using GeneSpring v.11.0.1 software (Agilent Technologies, Santa Clara, CA, USA). For qRT-PCR experiments, one treatment group comprised of 3–4 wells of Sc within one culture set. At least 3 such sets of cultures (performed on different calendar dates) were used to interpret the data. Data was expressed as mean ± SEM. Statistical analyses of data were performed by non-parametric student’s t-test for comparison between two groups. Statistical tests were done using GraphPad Prism 5.01 (GraphPad Software Inc., La Jolla, CA, USA). P values < 0.05 were considered as statistically significant.

## Results and discussion

This study was undertaken to determine the comprehensive gene expression profile of Sc obtained from different stages of postnatal testicular development. Sc drastically transform during pubertal maturation to fulfill the changing need of developing Gc. Therefore, the knowledge of differentially expressed genes by Sc during different postnatal ages will provide a deeper insight into Sc mediated regulation of spermatogenic onset at puberty and its maintenance in adulthood. Here, we have compared the differential gene expression of maturing (12 days old rats i.e. 12d) and mature (60 days old rats i.e. 60d) Sc, which have been normalized against that of the immature (5 days old rats i.e. 5d) Sc. Five-days-old (5d) neonatal rats represent proliferative Sc with the establishment of the spermatogonial stem cell niche on the basement membrane within the seminiferous epithelium; 12-days-old (12d) rats represent maturing Sc facilitating the robust generation of spermatogonia B [4,24] and 60-day-old rats (60d) represent fully mature, non-proliferative adult Sc where the blood testis barrier (BTB) is completely established with the presence of all stages of Gc upto sperm [18,24].

Microarray analyses have been employed by different laboratories to identify independent action of either FSH [25–27] or T [28–32] on testicular gene expression. Whereas both FSH and T act synergistically to regulate Sc gene expression for supporting Gc differentiation [2]. Therefore, major aim of the present study was to investigate the age related change in Sc transcriptome that has similar hormonal background. The doses of o-FSH and T were previously reported to be bioactive in terms of gene expression in Sc of all the age groups studied [4,18]. Similar to previous attempts [33,34], recently, Zimmermann and coworkers have reported the transcriptional dynamics of murine Sc revealing many candidate genes essential for the progression of first spermatogenic wave [35]. To understand if there are some common conserved genes between developing mouse and rat Sertoli cells, we have compared 394 differentially expressed genes obtained from Zimmermann’s data with 4395 genes found by us. We have identified 98 common genes which are accounted mostly for cell-cell communication, metabolism, energy production and cell growth (S1 Fig). However, it is important to note here that Zimmerman *et*.*al*. have reported Sc transcriptome at different postnatal ages in mouse, whereas our study distinctly illustrated rat Sc transcriptome under the influence of uniform hormonal stimulation.

Rat is considered a better model (than mice) for understanding the regulation of testicular functions [36]. Due to larger body size and higher blood volume and litter size, rats are more favored for surgical, physiological and pharmacological experiments [37,38]. Moreover, in terms of genetic distance, it is 5 million years closer to human than mice [39,40]. Keeping this in mind, rat Sc were used in the present study.

Previously flow sorted Sc were directly used for their RNA expression analyses [35]. Therefore, there is a probability that these cells may not be free from the proteolytic shock generated by the prolonged enzymatic exposure during the isolation process [41]. Such shock may induce transcriptional changes in the sorted cells contributing the final readout [42,43]. Therefore, in this study, instead of direct use of the isolated cells, Sc of all age groups were uniformly cultured for 4 days, a time period that was enough for stabilization the cells from the shock of the isolation process.

Pulsatile pattern of hormone release plays a critical role in optimal hormonal action in the target tissue as suggested by many *in vivo* studies [44,45]. The importance of pulsatile hormone treatment to endocrine cells has also been well-characterized in cultured pituitary cells [44] Administration of pulsatile GnRH (by changing both the amplitude and frequency) to cultured pituitary cells has been practiced for determining the differential transcriptional regulation of LH and FSH β subunits [44]. High and slow frequency pulses favor LH and FSH-β mRNAs expression, respectively [44]. Pulsatile release of gonadotropins is also known to regulate testicular function *in vivo* [46]. For example, pulsatile LH-releasing hormonal-therapy was found to be effective over constant GnRH in restoring the normal gonadotropin secretion inducing fertility in men with hypogonadotropic eunuchoidism [47]. We have recently demonstrated that for hormone induced gene expression study, pulsatile treatment of hormones (both FSH and T in combination) to cultured Sc from 18 days-old pre-pubertal rats’ shows better readouts as compare to that of the conventional, constant stimulation with hormones [48]. Although the establishment of hypothalamo-hypophyseal testicular axis steadily improves with age in rodents, GnRH pulse generator activity initiates during fetal development [49]. Moreover, neonatal Sc obtained from 5 days old rats do express both Androgen Receptor and FSH-Receptors [4]. Therefore, for the present study, cultured Sc of all three age groups were uniformly stimulated with pulsatile hormones (FSH and T in combination for ½ hr after every 3hr) on day 4 of culture to determine age specific, hormone induced differential transcriptome profile.

### Microarray analysis

**Fig 1:**
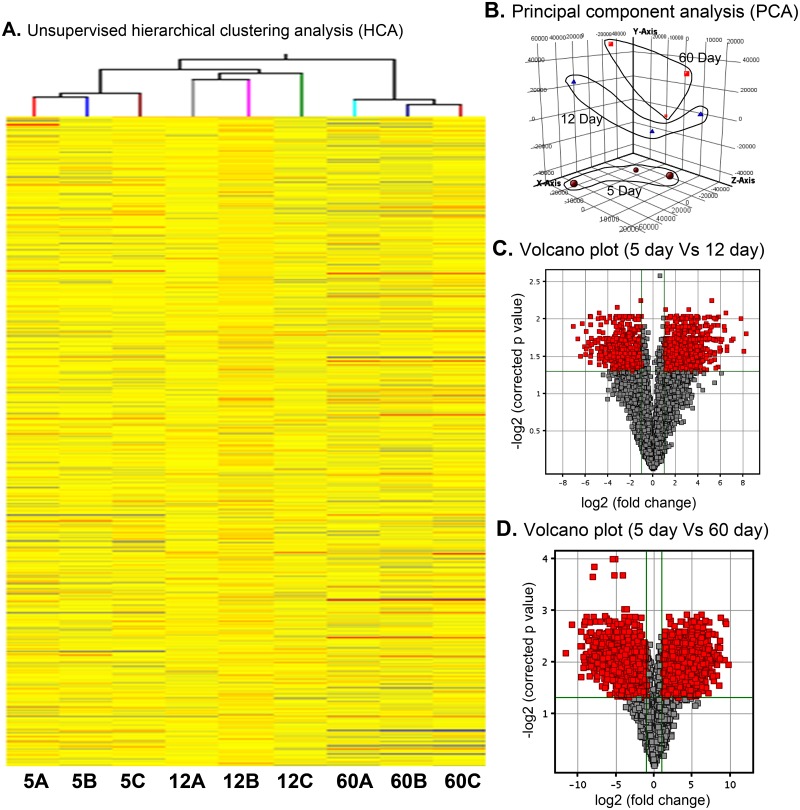
Analysis of microarray data for homogeneity of biological replicate samples. (**A**) Unsupervised hierarchical clustering analysis (**B**) Principal component analysis (**C**) Volcano plot analysis of differentially regulated genes between 5 days and 12 days and (**D**) Volcano plot analysis of differentially regulated genes between 5 days and 60 days.

RNA isolated from Sc was labeled with Cy3 and hybridized on Agilent’s rat whole genome 4x44K Gene Chips (Agilent Technologies, Santa Clara, USA). The results of unsupervised principal component analysis (PCA) of all samples and unsupervised hierarchic clustering analysis (HCA) revealed a high order of sample homogeneity. Fig 1A shows the results of the unsupervised HCA of the gene expression profiles of the 3 samples each of 5d, 12d and 60d Sc. The principal component analysis (PCA) shows that all the three biological replicates for 5d, 12d and 60d are falling in their respective dimensions (Fig 1B). Volcano plots of genes differentially expressed between 5d and 12d (Fig 1C) and 5d and 60d (Fig 1D) provides a visual presentation of fold change of genes is respect to p-value.

To identify the clusters of genes whose expression is regulated in a similar way throughout the samples, K-means clustering tool of GeneSpring software was applied to differentially expressed genes across the 3 samples each of 5d, 12d and 60d Sc. As shown in S2A Fig, 4 major groups (named as clusters 1, 2, 3 and 4) were identified. Gene Ontology (GO) based functionality analysis of K-mean clusters shows distribution of genes among cellular component (CC), molecular function (MF) and biological (BP) (S2B Fig). The raw microarray data has been submitted to NCBI GEO under the accession number GSE48795.

### Differential gene expression analysis

**Fig 2:**
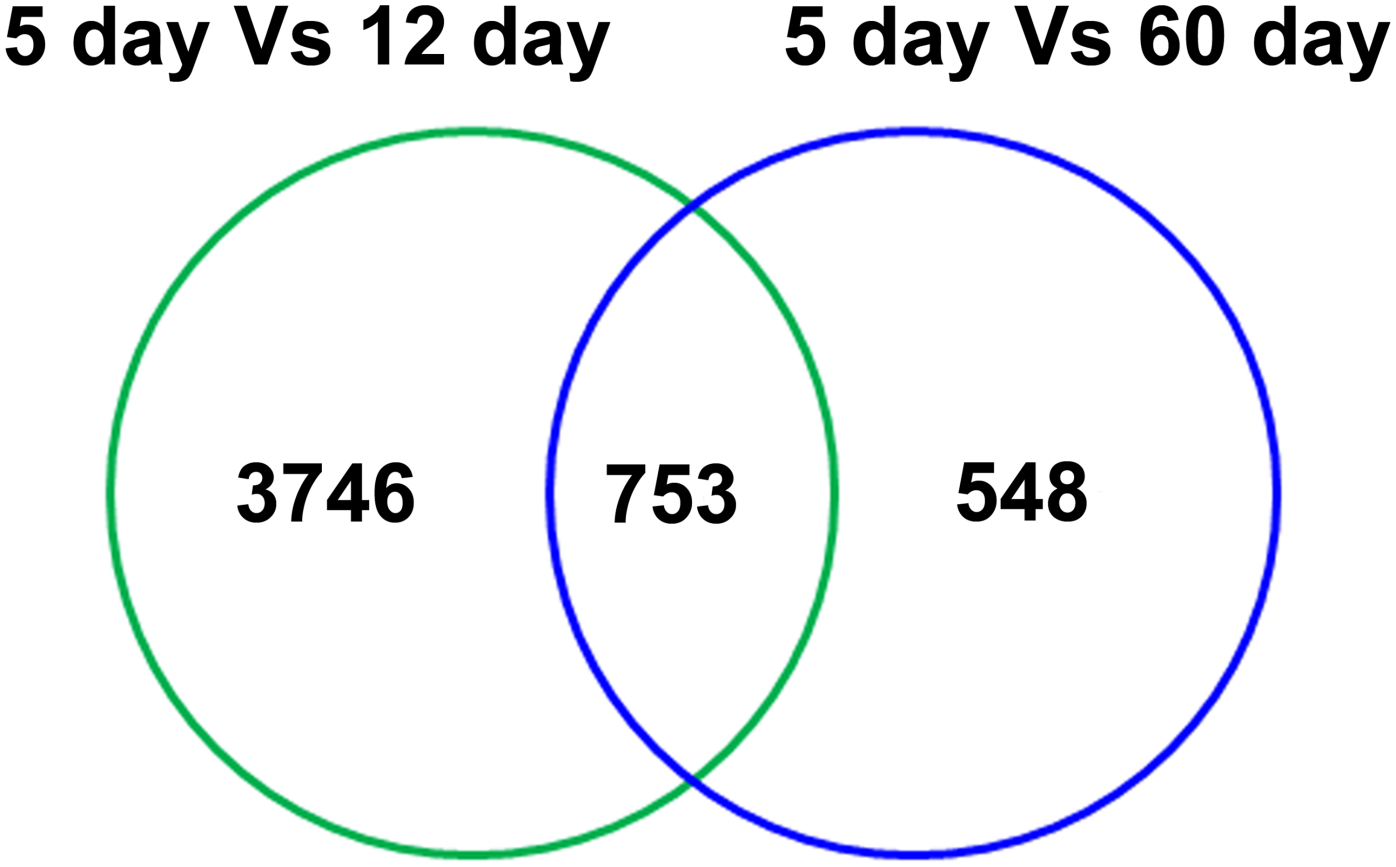
Commonality analysis of differentially regulated genes between 12 days and 60 days Sc in comparison with 5 days Sc.

The resulting gene lists from each pairwise comparison included the genes that showed a fold change of 2 (log_2_) or higher and a P < 0.05 by using a parametric Welch t test with Benjamini-Hochberg multiple testing corrections for false discovery rate (FDR). We compared the gene expression data obtained from12d and 60dSc against 5dSc to find differentially expressed genes. Venn diagram in Fig 2 shows that a total of 5074 genes were differentially regulated of which 3746 were exclusive in 12d and 548 were only in 60d. Out of 3746 total regulated genes in 12d, 1945 were upregulated and 1801 were downregulated (S1 and S2 Tables). Likewise, out of 548 genes regulated in 60d, 360 genes were upregulated and 188 genes were downregulated (S3 and S4 Tables). A possible explanation for this difference in the number of transcripts differentially regulated in 12d Sc and 60d Sc could be due to their respective developmental state. We have reported earlier that rat Sc acquire necessary developmental competence in terms of FSH responsiveness at 12 days of postnatal age coinciding with the rapid transition of spermatogonia A to B [4]. On the other hand, we have also reported recently that by 60 days of age Sc are fully differentiated and physiologically stable in terms of responsiveness towards T for supporting the spermatogenesis [18]. Therefore, it is reasonable to assume that such difference in the Sc physiology might be responsible for their differential gene expression pattern.

Some of the important and highly regulated genes upregulated in 12d, upregulated in 60d, downregulated in 12d, downregulated in 60d and differentially regulated in 12d and 60d are listed in Tables 2, 3, 4, 5 and 6, respectively.

**Table 2:** Genes upregulated in 12 days Sc.

Gene Symbol	Probe Set ID	Gene bank Accession	Gene Description	Expression Fold change
Tex101	A_44_P209196	NM_139037	Testis expressed gene 101	7.06
Sycp2	A_44_P185215	NM_130735	Synaptonemal complex protein 2	5.62
Sycp3	A_44_P459191	NM_013041	Synaptonemal complex protein 3	5.51
Ccnb1ip1	A_44_P901088	NM_001025141	Cyclin B1 interacting protein 1	5.48
Sycp1	A_43_P11573	NM_012810	Synaptonemal complex protein 1	4.76
Ddx4	A_42_P641569	NM_001077647	DEAD (Asp-Glu-Ala-Asp) box polypeptide 4	4.75
F2	A_44_P208138	NM_022924	Coagulation factor II	4.57
Slc30a2	A_42_P663722	NM_012890	Solute carrier family 30 (zinc transporter), member 2	4.57
Nr4a3	A_43_P12619	NM_031628	Nuclear receptor subfamily 4, group A, member 3	4.53
Stc1	A_44_P437956	NM_031123	Stanniocalcin 1	4.43
Edn1	A_44_P334736	NM_012548	Endothelin 1	4.39
Apoa4	A_44_P1030225	NM_012737	Apolipoprotein A-IV	4.35
Eaf2	A_42_P604033	NM_172047	ELL associated factor 2	4.31
Lgals4	A_42_P491119	NM_012975	Lectin, galactose binding, soluble 4	4.24

**Table 3:** Genes upregulated in 60 days Sc.

Gene Symbol	Probe set ID	Gene bank Accession	Gene Description	Expression fold change
Ldhc	A_44_P370052	NM_017266	Lactate dehydrogenase C	11.69
Fabp9	A_43_P12321	NM_022854	Fatty acid binding protein 9, testis	10.81
Aqp8	A_44_P244668	NM_019158	Aquaporin 8	9.66
Sycp3	A_44_P459191	NM_013041	Synaptonemal complex protein 3	9.64
Rpgrip1	A_44_P144765	NM_020366.3	Retinitis pigmentosaGTPase regulator interacting protein 1	9.23
Tex101	A_44_P209196	NM_139037	Testis expressed gene 101	9.07
Sycp1	A_43_P11573	NM_012810	Synaptonemal complex protein 1	8.99
Sycp2	A_44_P185215	NM_130735	Synaptonemal complex protein 2	8.94
Spetex-2D	A_44_P166068	NM_001011701	Spetex-2D protein	8.91
Crisp2	A_44_P237318	NM_031240	Cysteine-rich secretory protein 2	8.53

**Table 4:** Genes downregulated in 12 days Sc.

Gene Symbol	Probe set ID	Gene bank Accession	Gene Description	Expression fold change
MGC108747	A_44_P454376	NM_001009628	Similar to alpha-1 major acute phase protein prepeptide	-8.26
Kng1	A_44_P151156	NM_012696	Kininogen 1	-8.05667
Upk1b	A_43_P11086	NM_001024253	Uroplakin 1B	-6.98667
Agtr2	A_42_P470283	NM_012494	Angiotensin II receptor, type 2	-6.47
Fmo2	A_44_P486312	NM_144737	Flavin containing monooxygenase 2	-6.38
Cldn9	A_44_P419898	NM_001011889	Claudin 9	-6.34
Casp1	A_44_P468258	NM_012762	Caspase 1	-6.12333
Hoxa5	A_44_P262274	NM_019102.3	Homeobox protein Hox-A5	-6.07667
Itm2a	A_43_P10269	NM_001025712	Integral membrane protein 2A	-6.05
Spon1	A_43_P13420	NM_172067	Spondin 1	-5.90667
Mmp7	A_43_P11590	NM_012864	Matrix metallopeptidase 7	-5.88667

**Table 5:** Genes downregulated in 60 days Sc.

Gene Symbol	Probe set ID	Gene bank Accession	Gene Description	Expression fold change
Agtr2	A_42_P470283	NM_012494	Angiotensin II receptor, type 2	-9.44
Cdh22	A_44_P454259	NM_019161	Cadherin 22	-8.58
Krt2-7	A_44_P1029805	NM_001047870.1	keratin complex 2, basic, gene 7	-8.47
Cxcl12	A_44_P1034439	NM_022177	Chemokine (C-X-C motif) ligand 12	-8.32
Fmo2	A_44_P486312	NM_144737	Flavin containing monooxygenase 2	-7.90
Cdh11	A_43_P21114	NM_053392.1	Cadherin-11	-7.59
Pdgfra	A_42_P457003	M63837	Rat alpha-platelet-derived growth factor receptor mRNA	-7.56
Adcy2	A_43_P15311	NM_031007	Adenylate cyclase 2	-7.36
Sfrp2	A_44_P503115	NM_001100700.1	Putative secreted frizzled related protein	-7.22
Adamts5	A_44_P508162	NM_198761	A disintegrin-like and metallopeptidase (reprolysin type) with thrombospondin type 1 motif, 5 (aggrecanase-2)	-7.19

**Table 6:** Differential gene list.

Gene Symbol	Probe Set ID	Gene ID	Gene description	Expression (Fold Change) in comparison to 5 day	
				12 day	60 day
Cela1	A_44_P1050595	24331	chymotrypsin-like elastase family, member 1	-1.08	3.53
RT1-Db1	A_44_P130516	294270	RT1 class II, locus Db1	-1.04	4.69
Tekt2	A_42_P475233	298532	tektin 2 (testicular)	-1.29	3.75
Phf7	A_44_P1011595	364510	PHD finger protein 7	-1.03	5.49
Akr1c2	A_44_P323773	291283	aldo-keto reductase family 1, member C2	1.58	-2.86
Galntl5	A_44_P590256	499968	UDP-N-acetyl-alpha-D-galactosamine:polypeptide N-acetylgalactosaminyltransferase-like 5	-1.25	2.9
March11	A_44_P1023058	499558	membrane-associated ring finger (C3HC4) 11	-1.57	6.10
Ano1	A_42_P598679	309135	anoctamin 1, calcium activated chloride channel	-1.41	2.17
Npas2	A_44_P945456	316351	neuronal PAS domain protein 2	-1.38	2.17
Trim2	A_44_P980353	361970	tripartite motif-containing 2	1.04	-4.19

Gene Ontology (GO) terms analysis was performed using DAVID online resource. GO term analysis showed enrichment of relevant biological processes such as “Germ cell development”, “chemokine pathway”, “water transport”, “cell adhesion molecules”, “growth factors”, “growth factors binding proteins”, “insulin signaling”, “carbohydrate metabolism”, “lipid metabolic pathway”, “programmed cell death”, “cell division”, “differentiation” being the most prominent terms (Table 7).

**Table 7:** Functional category enrichment analysis based on gene ontology terms.

Biological Process	GO Term ID	Counts	% of Differentially Regulated genes	p-Value
Spermatogenesis	GO:0007283	19	7.8	4.1E-9
Microtubule cytoskeleton	GO:0015630	14	5.2	9.8E-4
Hexose biosynthetic process	GO:0019319	4	1.4	0.005
MAPK signaling pathway	GO:0051403	11	3.6	2.1E-7
Phosphate metabolic process	GO:0006796	107	7.6	7.6E-7
Neuron projection	GO:0043005	61	4.3	3.4E-6
Ion homeostasis	GO:0050801	58	4.1	8.3E-6
Cell adhesion	GO:0007155	64	4.5	1.08E-5
Cytoskeleton	GO:0005856	1.6	7.5	2.06E-5
Transcription regulator activity	GO:0030528	117	8.3	5.7E-5
Regulation of cell motion	GO:0051270	30	2.1	2.8E-4
Regulation of programmed cell death	GO:0043067	79	5.6	5.1E-4
Small GTPase mediated signal transduction	GO:0007264	33	2.3	9.2E-4
Growth factor binding	GO:0019838	11	3.6	2.6E-6
Vasculature development	GO:0001944	15	4.9	1.1E-4
Chemokine signaling pathway	GO:0070098	11	3.6	0.0011

% of DR refers to the percent of differentially regulated transcripts falling under the term; p-value is the raw p-value from Fishers exact test. GO term analysis was done using DAVID bioinformatics tool for functional analysis (http://david.abcc.ncifcrf.gov/).

From the differential gene expression profile list, our main focus was on the set of genes which might be important during postnatal maturation of Sc and therefore play crucial role in spermatogenesis. The gene clusters were selected on the basis of their respective relevance in spermatogenesis. After initial data analysis, a thorough literature search was done to understand how these gene clusters obtained in our microarray data were important in the process of spermatogenesis. Thereafter, the clusters of genes selected were used for further pathway analysis.

Genes potentially involved in some of the important biological functions related to spermatogenesis are discussed below.

### Chemokine signaling

**Fig 3:**
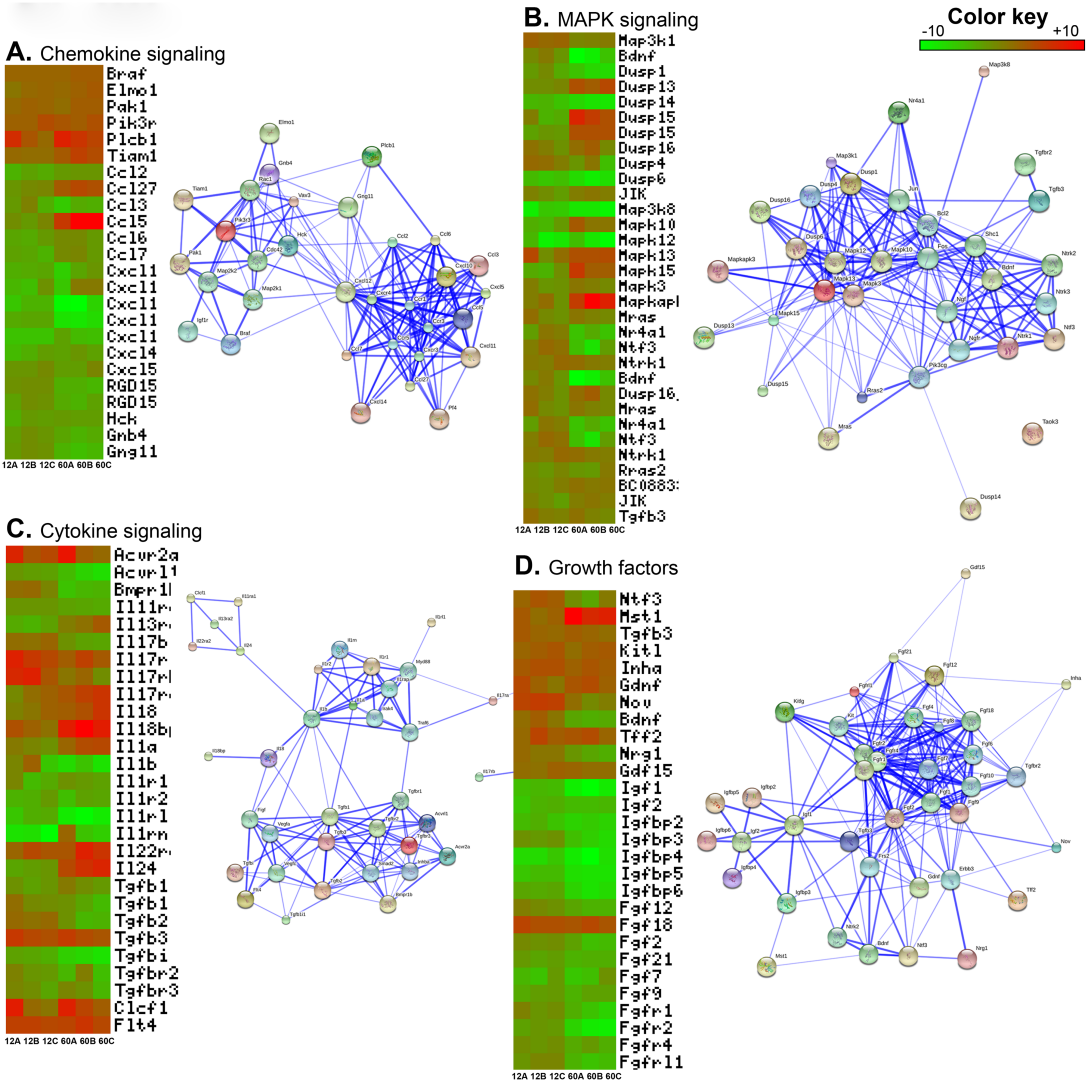
An overview of some important pathways differentially regulated in 12d and 60d Sc as compared to 5d Sc and their interactome map. Chemokine signaling (**3A**), MAPK signaling (**3B**), Cytokine signaling (**3C**) and Growth factors (**3D**). The heatmap shows differential expression of some selected pathways genes across three biological replicates in 12d and 60d Sc. Interacting network analysis of these genes shows how these genes interact with each other and form a network.

Chemokines are small cytokines important for chemotactic migration of cells including primordial germ cells. The chemokines that are important for homing of Gc, were downregulated in 12d and 60d Sc when Gc have already established in their respective niche. Since 5d Sc are actively involved in attracting Gc and creating germinal stem cell niche, higher expression of chemokine related genes by 5d Sc indicates important role of chemokine in immature testis (Fig 3A). Interacting network of these chemokine molecules reveals that chemokines and their receptors are closely related to each other. CXCL12, also known as stromal cell derived factor-1 (SDF-1), is expressed by Sc and CXCR4 is expressed by the Gc population of the adult human testes [50]. Interaction between the chemokine CXCL12 and its receptor CXCR4 is responsible for the maintenance of adult stem cell niches.

### Mitogen Activated Protein Kinase (MAPK) pathway

The MAPK pathway acts to integrate diverse signals to regulate a variety of cellular functions such as cell cycle progression, cell adherence, motility and metabolism and thereby influence a number of developmental processes [51]. MAPK pathway is pre-dominant in immature Sc and downregulate upon maturation of Sc [52]. MAPK pathway genes were differentially expressed in microarray data. Majority of these genes were downregulated in both 12d and 60d Sc. All of these genes were directly interacting with each other forming strong network (Fig 3B). Genes which were downregulated in 60d but induced in 12d Sc were dual specificity phosphatases (Dusp1&7), brain derived neurotrophic factor (Bdnf), nuclear receptor subfamily 4, group A, member 1 (Nr4a1) and neurotrophin 3 (Ntf3). MAP3K1 act to regulate or integrate signals during testis development as Map3k1-deficient mice are non-viable [53]. p38 MAPK signaling initiates G1 mitotic arrest in a variety of terminally differentiated cell types [54]. DUSP and JIK were upregulated in 12d and 60d Sc. [55,56]. These two genes are negative feedback regulator of MAPK signaling, suggesting predominant role of MAPK signaling is immature Sc but not upon their differentiation [57].

### Cytokine signaling

Cytokines are potent growth factors and their differential expression is important for regulating different functions in infant and adult testis [58]. Sc produce cytokines such as Interleukins (ILs), Interferons (IFNs), Tumor necrosis factor (TNF), transforming growth factor (TGF) etc. which play important role in spermatogenesis [59]. Interleukins were the majority of cytokines found differentially expressed in our microarray data. With exception of few, most of these IL, their isoforms and their receptors were downregulated in both 12d and 60d Sc. (Fig 3C). The Transforming Growth Factor beta (TGFβ) superfamily of ligands, including TGFBs, activins, bone morphogenetic proteins (BMPs), nodal, and growth and differentiation factors (GDFs) govern Gc development [60]. TGF-b3 regulates the permeability of Sc tight junctions helping spermatogonia to cross BTB during progression of spermatogenesis and release of mature sperm while spermiation [61,62, 63]. IL-1α is the most abundant Sc growth factor in the pubertal and adult testis while IL-1β is a potent growth factor for immature rat Sc [64]. Activin regulate number of Gc in the fetal testis and Gc maturation at the onset of spermatogenesis, contributing to the complex signaling networks that govern normal testis development [65]. Network analysis shows that interleukins and transforming growth factor molecules make two distinct networks which are connected to each other.

### Growth factors

A number of growth factors are expressed in testis which control migration, differentiation, and proliferation of primordial germ cells and Sc [66]. Majority of these growth factors are Insulin-like growth factors, Fibroblast growth factors and their receptors and binding proteins. Growth factors provide signals for developing Sc and hence their expression is increased in 12d Sc. Elevated expression of these genes up to 12d of age in Sc signifies their role in migration of Gc in SSC niche, proliferation of spermatogonia, entry of spermatogonia in meiosis and Sc-Gc interaction. Some genes i.e. PDGFa, Mst1, KitL, GDNF, TFF2 and GDF15 were induced in 12d Sc and were persistently expressed in 60d Sc also (Fig 3D). These genes interact with each other and forms strong network. These genes are implicated in proper germ-line development and regulation of proliferation and differentiation of Gc during spermatogenesis [66,67]. Genes of the IGF signaling pathway has been shown to express in testis, especially in Sc. They act as strong mitogenic and anti-apoptotic signal during proliferation of Sc. IGF1 inhibits differentiation of Gc [68]. FGFs are involved in the proliferation and differentiation of testicular cells and involved in the regulation of spermatogenesis [69]. In contrast to other growth factors, FGF18 was upregulated in both 12d and 60d Sc. FGF18 plays role in pre-Sc functions but its role after birth has yet not been assigned.

### Small GTPases and cytoskeleton maintenance

**Fig 4:**
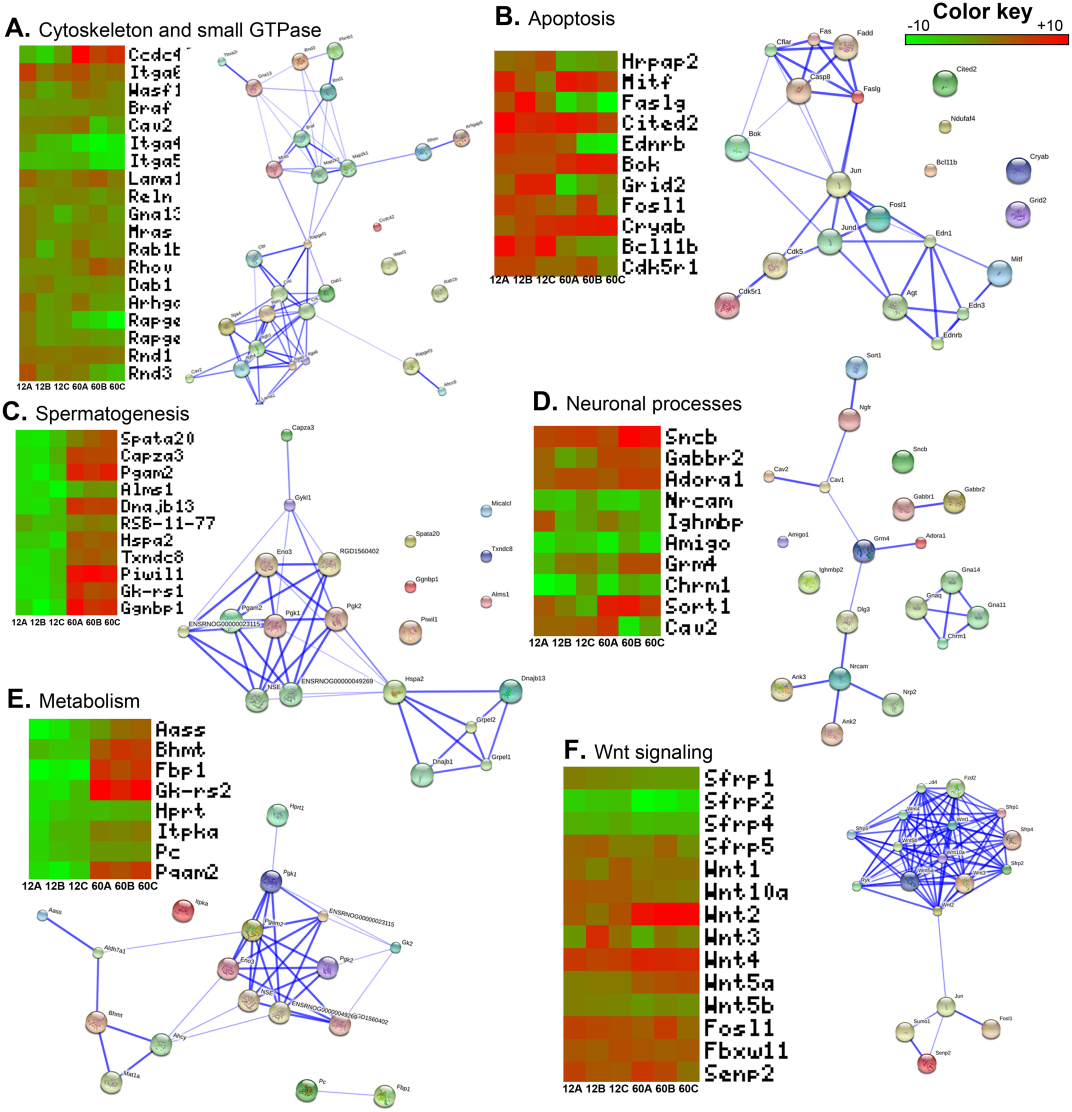
An overview of some important pathways differentially regulated in 12d and 60d Sc as compared to 5d Sc and their interactome map. Cytoskeleton and small GTPases (**4A**), Apoptosis (**4B**), Spermatogenesis (**4C**), Neuronal processes (**4D**), Metabolism (**4E**) and Wnt signaling (**4F**). The heatmap shows differential expression of some selected pathways genes across three biological replicates in 12d and 60d Sc. Interacting network analysis of these genes shows how these genes interact with each other and form a network.

Small GTpases are involved in regulating Sc-Gc tight junctions, Gc movement, and cell division [62]. Small GTPase which are upregulated in both 12d and 60d Sc were guanine nucleotide binding protein, alpha 13 (Gna13), muscle and microspikes RAS (Mras), Ras-related protein Rab-1B (Rab1b), ras homolog gene family, member V (Rhov), disabled homolog 1 (Dab1) and Rho GTPase activating protein 5 (Arhgap5). Members of Rho GTPases are important part of the signaling pathway that is involved in inducing cytoskeletal changes, lamellipodia and filopodia formation and the promotion of cell motility [70]. Activation of RhoBGTPases modulates the actin cytoskeleton network resulting in adjacent junction disruption and germ cell loss from the seminiferous epithelium [62]. Sc have a highly developed membrane trafficking system that might be regulated by small GTPases [71] (Fig 4A). Network analysis reveals that these genes make two networks which are interconnected.

Many genes involved in cell-cell junctions were found well expressed in 12d and 60d Sc. These genes such as Integrin alpha 6 subchain (Itga6), vinculin (Vcl), V-raf murine sarcoma viral oncogene B1-like protein (Braf), laminin, alpha 1 (Lama1), Collagen alpha 2 (Cola2) and reelin (Reln) forms important interacting component of Sc-Sc and Sc-Gc junctions. We found Integrin alpha 4 subchain (Itga4) and Integrin alpha 5 subchain (Itga5) downregulated in both 12d and 60d Sc. These two genes are reported to be highly expressed in embryonic and infant testis as they are crucial for making spermatogonial stem cell niches [70].

### Apoptosis and phagocytosis

Many of the genes involved in programmed cell death were induced in 12d Sc. The genes upregulated in both 12d and 60d were Microphthalmia-associated transcription factor (Mitf), Cbp/p300-interacting transactivator, with Glu/Asp-rich carboxy-terminal domain, 2 (Cited2), Bcl-2-related ovarian killer protein (Bok), crystallin, alpha B (Cryab). Fos-like antigen 1 (Fosl1) and cyclin-dependent kinase 5, regulatory subunit 1 (p35) (Cdk5r1) were upregulated in 12d but remain unchanged in 60d Sc. The genes which were upregulated in 12d but downregulated in 60d were hormone-regulated proliferation associated protein 20 (Hrpap20), Fas ligand (TNF superfamily, member 6) (Faslg), endothelin receptor type B (Ednrb), glutamate receptor, ionotropic, delta 2 (Grid2) and similar to zinc finger protein mRit1 beta (Bcl11b) (Fig 4B). Many of the genes in this pathway are connected with each other in a network. However, this network is not very strong as compared to other networks.

Genes involved in phagocytosis were dynein, cytoplasmic, heavy polypeptide 2 (Dnch2), cartilage oligomeric matrix protein (Comp), mannose receptor, C type 1 (Mrc1) and phospholipase A2 receptor (Pla2r1). These genes were upregulated in 60d Sc. These genes were either slightly downregulated or remained unaltered in 12d Sc.

### Genes involved in homeostasis

Another major group of genes conspicuously expressed by mature Sc are the genes which are important for maintenance of homeostatic environment inside seminiferous tubule. This included genes from sodium and potassium ion channels, water transporters, voltage gated ion channels, voltage dependent chloride channels etc. Sc play a key role in the establishment of an adequate luminal environment in the seminiferous tubules of the male reproductive tract. Secretion of the seminiferous tubular fluid (STF) is important for successful completion of spermatogenesis as it provides the medium of transport to maturing spermatozoa [72]. STF release begins during sexual development and is dependent on FSH [73,74]. Sc regulate the passage of ions and the selective flow of water, steroids and carbohydrates into the tubular lumen [75].

### Spermatogenesis

The genes which are important for maturation of sperm in adult were found to upregulated in 60d Sc. The example of some of these genes are Spermatogenesis Associated 20 (Spata20), Capping Actin Protein Of Muscle Z-Line Alpha Subunit 3 (Capza3), Phosphoglycerate Mutase 2 (Pgam2), DnaJ Heat Shock Protein Family (Hsp40) Member B13 (Dnajb13), Heat Shock Protein Family A (Hsp70) Member 2 (Hspa2), Thioredoxin Domain Containing 8 (Txndc8), P-element Induced WImpy testis in Drosophila 11 (Piwi11), glycerol kinase-like 1 (GK-rs1) and Gametogenetin Binding Protein 1 (Ggnbp1) (Fig 4C).

### Genes expressed in nervous system

The genes which are normally expressed in neuronal processes were found to be expressed by Sc. Interestingly, genes which are prominently expressed in nervous system were found upregulated in 12d and 60d Sc. Sortilin1 (Sort1) is expressed in brain, spinal cord and muscles but also has roles in embryogenesis [76–78]. It acts as receptor for neurotensin and co-receptor for Nerve Growth Factor (NGF) and Brain Derived Neurotrophic Factor (BDNF) mediating cell survival and apoptosis. Nerve growth factors have been shown to play important role in Sc-Sc interaction [79,80]. Caveolae are small invaginations of the plasma membrane which plays important roles in signal transduction, cholesterol transport, and endocytosis [81,82]. Caveolin2 are more abundant in terminally differentiated cells [83]. Most of such genes were upregulated in both 12d and 60d Sc. (Fig 4D).

### Metabolism

Sc possess specialized metabolic mechanisms to meet specific energy demand of developing germ cells [84]. Sertoli cells convert glucose into lactate which is preferentially uptake up by germ cells. In our microarray results, most of the genes which are associated with glucose metabolism were induced in 60 day Sc. These genes were either remained unaltered or slightly downregulated in 12d Sc. The example of such genes are aminoadipate-semialdehyde synthase (Aass), betaine-homocysteine methyltransferase (Bhmt), glucokinase activity, related sequence 2 (Gk-rs2), hypoxanthine guanine phosphoribosyl transferase (Hprt), inositol 1,4,5-trisphosphate 3-kinase A (Itpka), Pyruvate carboxylase (Pc) and phosphoglycerate mutase 2 (Pgam2). fructose-1,6- biphosphatase 1 (Fbp1) was downregulated in 12dSc (Fig 4E).

### Wnt signaling

The wingless-related MMTV integration site (WNT) gene family encodes a large number of secreted signaling glycoproteins that are involved in many biological processes including embryonic development, adult tissue homeostasis, maintenance of progenitor cell types and cell fate determination and differentiation [85]. Differential expression of Wnt signaling is important for proper functioning as Sc specific constitutive activation of WNT/CTNNB pathway causing sterility. Wnt ligands (Wnt4, Wnt5a) were upregulated and Wnt pathway inhibitors (sFRP) were downregulated in 12d and 60d Sc. Wnt 4 is important for proper functioning of Sc after birth as differentiation of Sc becomes compromised in Wnt4 mutant testes [86]. There are no reports of Wnt 2 expression in Sc hence upregulation of Wnt 2 in only 60d Sc may be intriguing. WNT5a has been shown to promote SSC self-renewal [87]. Secreted Frizzled-related proteins (sFRPs) are secreted glycoproteins that can antagonize WNT mediated signaling by direct competitive interaction with WNT ligands [88,89]. sFRP1 has been shown to downregulate in mature testis showing its importance in regulating spermiation in adult rat testis [90] (Fig 4F). Network analysis reveals that all the Wnt ligands and receptors form strong network and are closely related. The other members of the pathway form another network and both these networks are connected together.

### qRT-PCR validation of genes identified by microarray

**Fig 5:**
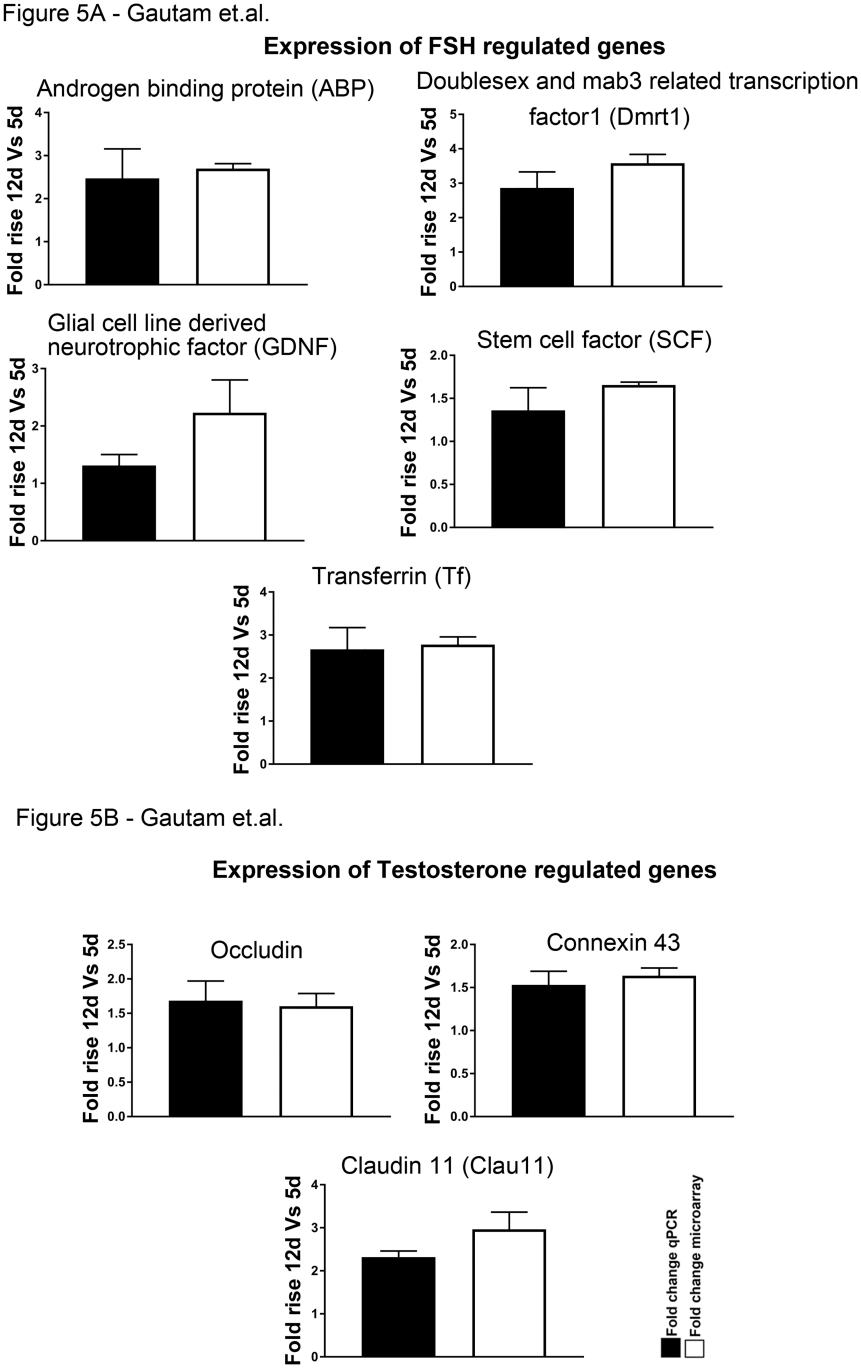
Fold rise in FSH (A) and T (B) responsive genes compared between microarray data and Quantitative real time PCR. Data represented as mean ± SEM.

**Fig 6:**
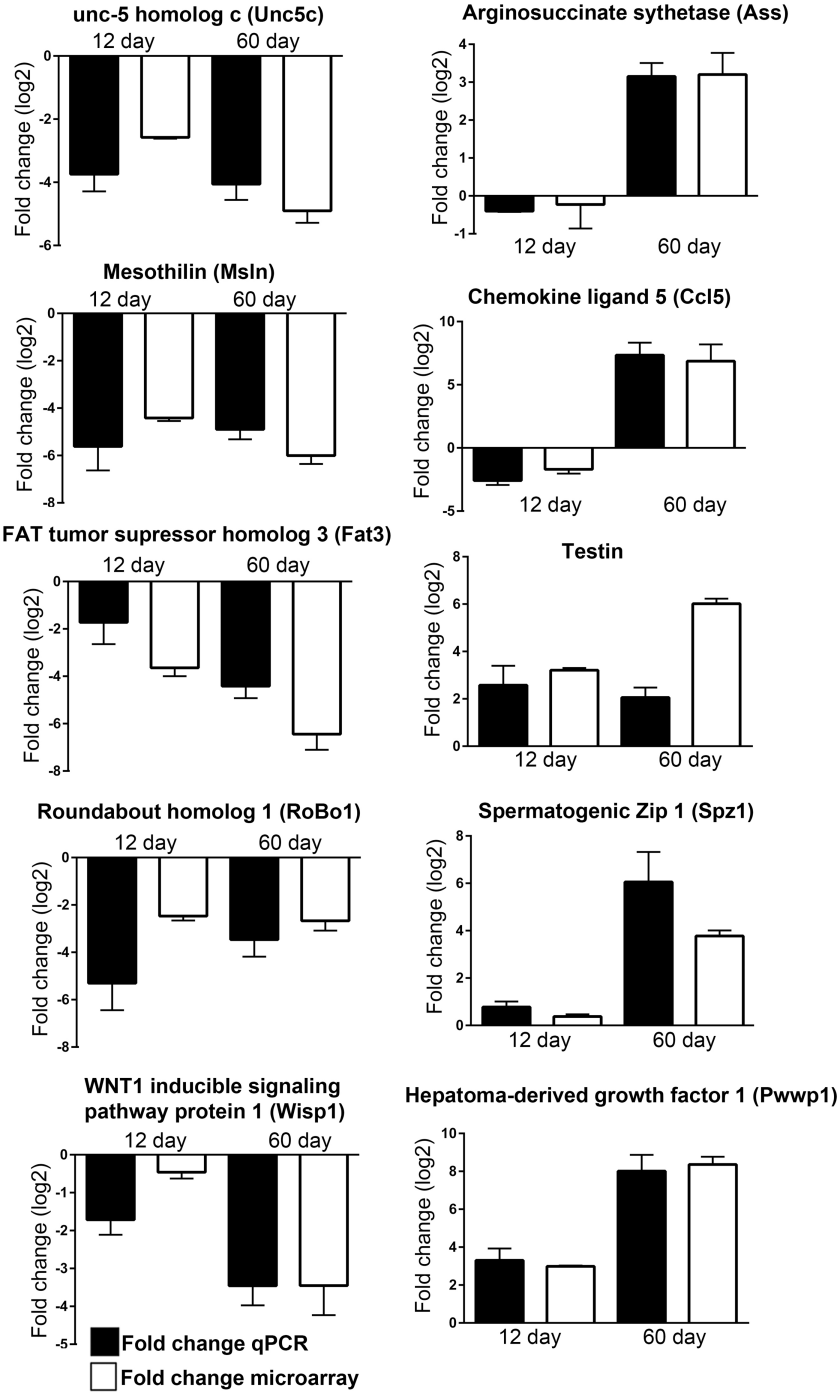
Validation of some selected genes from microarray data using Quantitative real time PCR. Data represented as mean ± SEM.

In order to authenticate the array data the expression patterns of some of the genes were validated by quantitative reverse transcription PCR (q-RT-PCR) with additional sets of Sc culture obtained from all three age groups with consistent hormonal treatments (pulsatile FSH and T in combination for 11hr) and were compared with that of the array data. The genes considered were divided in five groups: 1) FSH responsive genes like SCF, GDNF, ABP, Transferrin and Dmrt1 in both 5d and 12d as reported earlier either by us or others [2–4]. T responsive genes like Claudin11, Occludin and Connexin43 in both 5d and 12d as reported earlier either by us or others [3,4,91]. Genes downregulated in both 12d and 60d [UNC-5 family of netrin receptors (Unc5c), Mesothelin (Msln), FAT Tumor Suppressor Homolog 3 (Fat3), Roundabout homolog 1 (Robo1) and WNT1-inducible-signaling pathway protein 2 (Wisp)]; 2) genes downregulated in 12d but upregulated in 60d [Alpha-aminoadipic semialdehyde synthase (Aass), Chemokine (C-C motif) ligand 5 (Ccl5)], and 3) genes upregulated in both 12d and 60d [Hepatoma derived growth factor 1 (Pwwp1), spermatogenic zip 1 (Spz 1) and Testin]. For each sample, the calculated quantity of each gene was normalized with the endogenous control Ppia (Cyclophilin A). The relative quantities of mRNAs for all target genes were determined by 2^-ΔΔCt^ method as reported by us before [4,19]. It is important to note that, like the array data, the gene expression values obtained from 12d and 60d Sc by q-RT-PCR were normalized against that found in 5d Sc. Expression patterns of both FSH or T responsive genes (Fig 5A and 5B) and some other selective genes (Fig 6) generated by qRT-PCR analyses were consistent with their expression pattern found in microarray. This observation further strengthens the authenticity of our array data.

## Conclusion

Postnatal maturation of testicular Sc is critical in regulating male fertility. Therefore, it becomes essential to investigate the genes expressed by Sc during this phase of development. Our microarray results have indicated differential expression of genes associated with cyto-architecture, metabolism, cytokines, chemokines, growth factors, MAPK signaling and Wnt signaling among others. Taken together, this differential transcriptome data provide an important resource database to reveal the molecular network of Sc maturation which is necessary to govern male germ cell differentiation. This information will improve our current understanding of the etiology of some forms of idiopathic male infertility with persistent immature Sc even in adulthood.

## Supporting information

S1 Fig Comparison of gene sets between the present study and the data published by Zimmerman et.al. There are 96 genes common between our data and Zimmerman’s data and these genes are mainly involved in signal transduction, cell-cell communication, energy and metabolism and cell growth.
(TIF)

S2 Fig K-mean analysis of co-expressed genes. (**A**) Entity based clustering analysis of co-expressed genes identified four clusters of co-expressed genes. (**B**) Gene Ontology (GO) based functional analysis of genes grouped under four clusters. Functional analysis was performed for molecular function (MF), cellular components (CC) and biological processes (BP).
(TIF)

S1 Table Genes upregulated in 12d Sc. (XLS)

S2 Table Genes downregulated in 12d Sc. (XLS)

S3 Table Genes upregulated in 60d Sc. (XLS)

S4 Table Genes downregulated in 60d Sc. (XLS)
