# Supplementary figures and images for: Hormone induced differential transcriptome analysis of Sertoli cells during postnatal maturation of rat testes
Source: PLoS One. 2018 Jan 17;13(1):e0191201. doi: 10.1371/journal.pone.0191201 (PMC5771609; doi:10.1371/journal.pone.0191201)

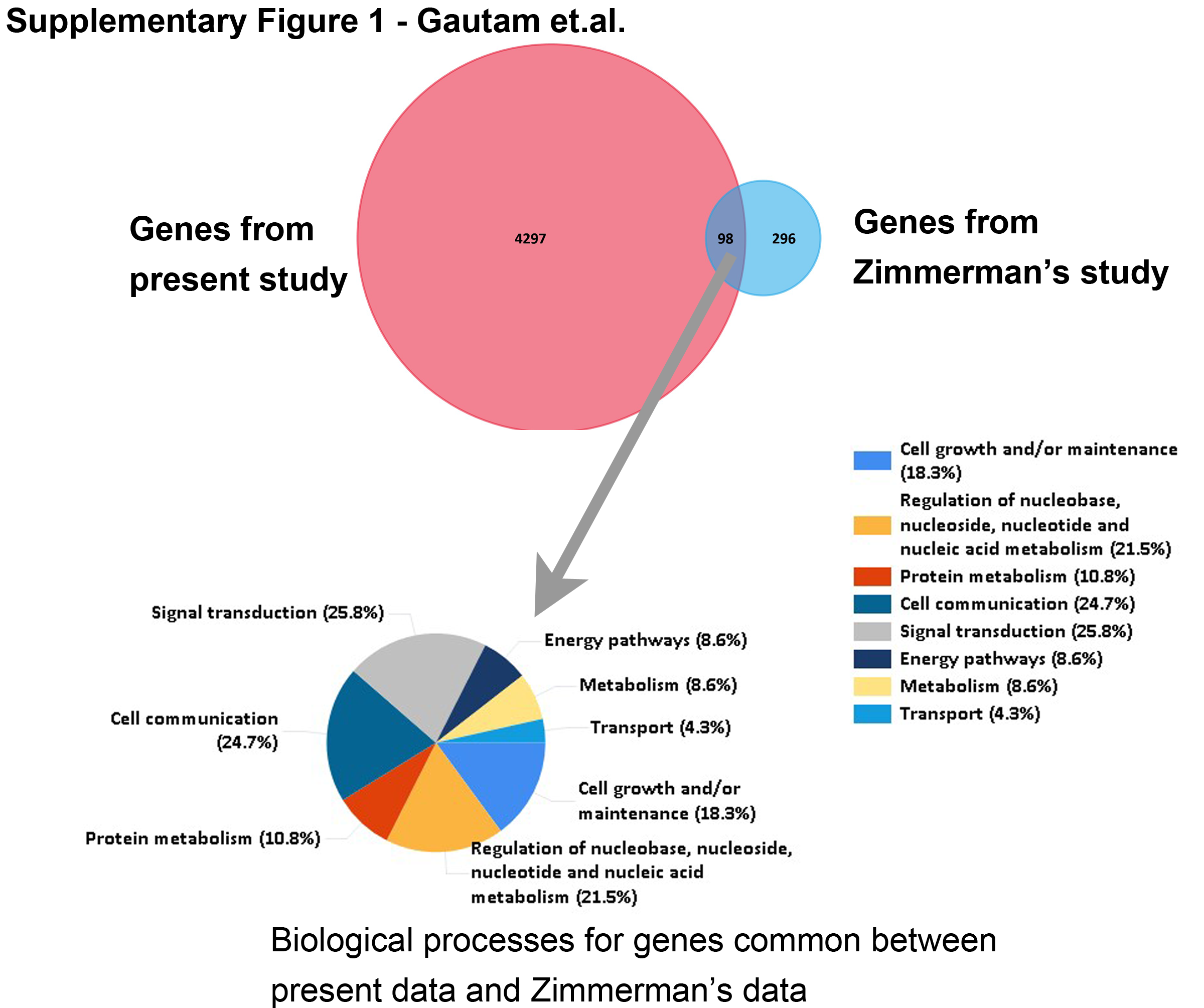

Supplement: S1 Fig — There are 96 genes common between our data and Zimmerman’s data and these genes are mainly involved in signal transduction, cell-cell communication, energy and metabolism and cell growth. (TIF) [file pone.0191201.s001.tif]

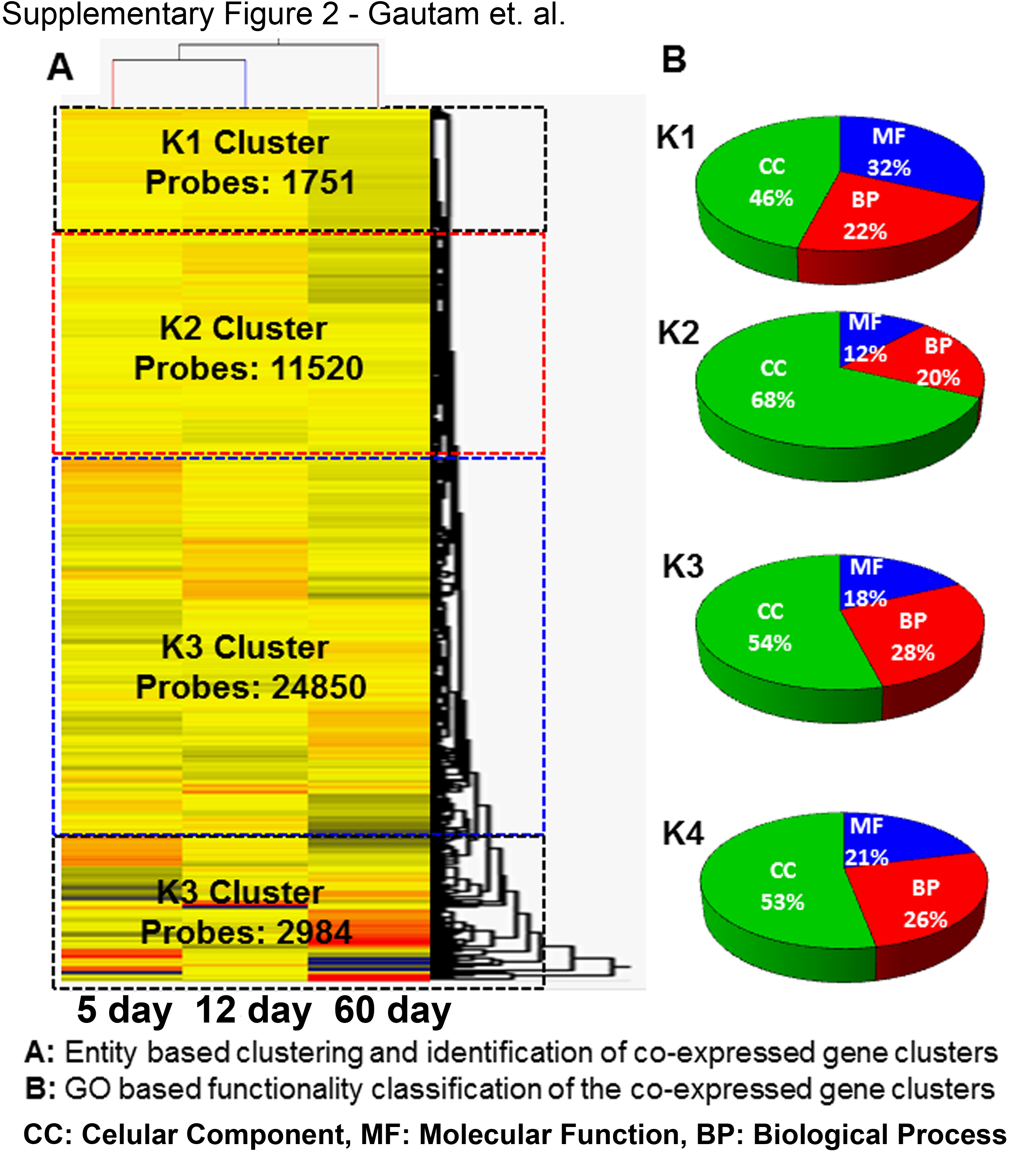

Supplement: S2 Fig — (A) Entity based clustering analysis of co-expressed genes identified four clusters of co-expressed genes. (B) Gene Ontology (GO) based functional analysis of genes grouped under four clusters. Functional analysis was performed for molecular function (MF), cellular components (CC) and biological processes (BP). (TIF) [file pone.0191201.s002.tif]
